# Acute Systolic Heart Failure Associated with Complement-Mediated Hemolytic Uremic Syndrome

**DOI:** 10.1155/2015/327980

**Published:** 2015-10-18

**Authors:** John L. Vaughn, Jared M. Moore, Spero R. Cataland

**Affiliations:** ^1^Department of Internal Medicine, The Ohio State University, 395 W. 12th Avenue, Third Floor, Columbus, OH 43210, USA; ^2^Division of Hematology, The Ohio State University, 320 W. 10th Avenue, Columbus, OH 43210, USA

## Abstract

Complement-mediated hemolytic uremic syndrome (otherwise known as atypical HUS) is a rare disorder of uncontrolled complement activation that may be associated with heart failure. We report the case of a 49-year-old female with no history of heart disease who presented with microangiopathic hemolytic anemia, thrombocytopenia, and acute kidney injury. Given her normal ADAMSTS13 activity, evidence of increased complement activation, and renal biopsy showing evidence of thrombotic microangiopathy, she was diagnosed with complement-mediated HUS. She subsequently developed acute hypoxemic respiratory failure secondary to pulmonary edema requiring intubation and mechanical ventilation. A transthoracic echocardiogram showed evidence of a Takotsubo cardiomyopathy with an estimated left ventricular ejection fraction of 20%, though ischemic cardiomyopathy could not be ruled out. Treatment was initiated with eculizumab. After several failed attempts at extubation, she eventually underwent tracheotomy. She also required hemodialysis to improve her uremia and hypervolemia. After seven weeks of hospitalization and five doses of eculizumab, her renal function and respiratory status improved, and she was discharged in stable condition on room air and independent of hemodialysis. Our case illustrates a rare association between acute systolic heart failure and complement-mediated HUS and highlights the potential of eculizumab in stabilizing even the most critically-ill patients with complement-mediated disease.

## 1. Introduction

The hemolytic uremic syndrome (HUS) is a rare syndrome that is characterized by microangiopathic hemolytic anemia, thrombocytopenia, and acute kidney injury [1]. HUS is divided into two broad categories according to its underlying pathophysiology: primary complement-mediated HUS (otherwise known as atypical HUS) and secondary HUS [1]. Primary complemented-mediated HUS is caused by uncontrolled complement activation secondary to mutations of complement proteins, gain-of-function mutations that render complement proteins less susceptible to inactivation, or autoantibodies that develop against complement proteins [1]. Secondary HUS is usually caused by infection due to Shiga toxin-producing* E. coli*, but it may also be due to other types of infection, drugs, pregnancy, or autoimmune disorders [1]. In all types of complement-mediated HUS, uncontrolled complement activation leads to the formation of the membrane attack complex (MAC), endothelial damage, and activation of the coagulation cascade. It is important to differentiate HUS from thrombotic thrombocytopenia purpura (TTP), which is caused by deficient activity of the enzyme protease ADAMTS13 [2, 3].

We report the outcome of a patient who presented with complement-mediated HUS and developed acute systolic heart failure. She was treated with eculizumab and recovered after an extended hospitalization.

## 2. Case Report

A 49-year-old female with no history of heart disease presented to the emergency department with a 10-day history of intermittent nausea, vomiting, and diarrhea. Her laboratory findings were significant for a hemoglobin of 8.7 mg/dL, platelets of 46 × 10^3^/*μ*L, serum creatinine of 2.36 mg/dL, lactate dehydrogenase of 1030 U/L, haptoglobin <6 mg/dL, and a reticulocyte count of 5.1%. Her peripheral blood smear showed rare schistocytes. Given the concern for HUS or TTP, a central venous catheter was inserted and plasma exchange was initiated. However, her ADAMTS13 activity was subsequently found to be >100% and plasma exchange was stopped after two treatments. She was also found to have C3 of 54 mg/dL (normal range: 80–178 mg/dL), C4 of 7 mg/dL (normal range: 12–42 mg/dL), Bb of 8039 ng/mL (normal range: 244–961 ng/mL), and C5b-9 (MAC) of 570 ng/mL (normal range: 34–248 ng/mL). A renal biopsy showed evidence of thrombotic microangiopathy. Consequently, she was diagnosed with complement-mediated HUS.

Before treatment could be initiated with eculizumab, she developed acute hypoxemic respiratory failure secondary to pulmonary edema requiring endotracheal intubation and mechanical ventilation. She was transferred to the medical intensive care unit where she was started on renal replacement therapy for uremia and hypervolemia. A transthoracic echocardiogram showed severe segmental left ventricular dysfunction in a distribution that was consistent with a Takotsubo cardiomyopathy with an estimated left ventricular ejection fraction of 20%, though ischemic cardiomyopathy could not be ruled out. Given her acute kidney injury, cardiac catheterization was not performed. After receiving meningococcal vaccination, she was started on eculizumab at a dose of 900 mg weekly for four weeks, then 1200 mg every other week starting at week five. Multiple attempts were made to extubate her, but she eventually required tracheotomy for ventilator weaning. Her hospitalization was further complicated by renal biopsy-associated perinephric hematoma that resulted in hemorrhagic shock. This was treated with renal artery embolization.

After seven weeks of hospitalization and five doses of eculizumab, her renal function and respiratory status improved, and she was discharged in stable condition on room air and independent of hemodialysis.  Table 1 illustrates the changes in her platelets, serum creatinine, and lactate dehydrogenase that occurred during her hospitalization. Following discharge, genetic analysis subsequently revealed genetic variants of C3 and complement factor I (CFI). Her C3 gene had a c.463A>C, p.Lys155Gln variant and her CF1 gene had a c.1657C>T, p.Pro553Ser variant. She tested negative for complement factor H autoantibodies. One month after her original hospitalization, she was admitted again for a heart failure exacerbation. She was treated with diuretics for one week and discharged again in stable condition. A repeat transthoracic echocardiogram obtained one month after her second hospitalization showed improvement in her estimated left ventricular ejection fraction to 40–45% without the wall motion abnormalities visualized previously. Twelve weeks after her second hospitalization, her terminal complement levels remained abnormal with a Bb of 1416 ng/mL and a C5b-9 of 300 ng/mL. However, she returned to her previous level of functioning and continued to receive eculizumab as an outpatient.

## 3. Discussion

Acute heart failure is a potentially fatal complication of thrombotic microangiopathies with an estimated incidence of 9.5% in one single institutional case series [4]. However, the incidence and prognosis of acute heart failure in patients with complement-mediated HUS remain unknown, and case reports of acute heart failure associated with complement-mediated HUS are rare. Our case report provides evidence that patients with complement-mediated HUS and acute heart failure treated with eculizumab may recover cardiac function over time. Although our patient decompensated the requiring transfer to the medical intensive care unit and a prolonged hospitalization, her cardiac function gradually improved and she returned to her previous functional state. Her slow recovery despite eculizumab therapy was likely secondary to her acute heart failure.

The pathophysiology of acute heart failure in patients with thrombotic microangiopathies remains undefined. Multiple mechanisms have been proposed including high output heart failure secondary to anemia and myocardial ischemia secondary to microvascular thrombi [4, 5]. In complement-mediated HUS, increased complement activation interferes with endothelial resistance to thrombosis, which may contribute to the formation of microvascular thrombi [6]. Our patient had evidence of a Takotsubo cardiomyopathy, which is a poorly understood cardiomyopathy that often presents with signs and symptoms of myocardial infarction without demonstrable obstructive coronary artery disease. Takotsubo cardiomyopathy may be partly due to microvascular dysfunction [7]. Although we were not able to rule out obstructive coronary artery disease with cardiac catheterization, our patient had no risk factors for this.

Our patient had mutations in her C3 and CFI genes. Both variants have been reported previously and may have predisposed her to complement-mediated HUS, though the pathogenic significance of those variants remains uncertain [8–10]. The C3 gene encodes the C3 protein, which plays a central role in the complement system. Gain-of-function mutations in the C3 gene are known to cause complement dysregulation [10], and there is evidence that patients with C3 mutations are more likely to develop cardiovascular complications [6]. The CFI gene encodes the complement factor I protein, which regulates complement activation by cleaving other complement proteins. Loss-of-function mutations in complement factor I are known to cause complement dysregulation [10]. It is possible that a trigger event such as gastrointestinal infection precipitated complement-mediated HUS in our patient because she was genetically predisposed to the disease. Our patient had abnormal levels of her serum complements, but patients with other mutations may have normal levels of normal levels of C3, C4, CFB, CFH, and CFI [11].

Finally, our case highlights the potential of eculizumab in stabilizing even the most critically-ill patients with complement-mediated HUS. Eculizumab is a humanized monoclonal IgG antibody that binds to the C5 protein and prevents the formation of the terminal complex C5b-9 (MAC) [12]. Our findings are consistent with two prospective phase 2 trials that demonstrated dramatic improvement in patients with complement-mediated HUS treated with eculizumab [12]. Initiation of eculizumab in our patient resulted in time-dependent improvement in cardiac, hematologic, and renal parameters. Although increasing evidence indicates that eculizumab may be discontinued in some cases [13], the persistent terminal complement activation in our patient suggests that ongoing therapy is needed.

## Figures and Tables

**Table 1:** Platelets, serum creatinine, and lactate dehydrogenase measurements from day 1 of initial hospitalization until discharge. Plasma exchange was initiated on day 1 and stopped on day 2. Eculizumab was initiated on day 12. Renal replacement therapy was initiated on day 16 and stopped on day 46.

Day	Platelets	Serum creatinine	Lactate dehydrogenase
	(×10^3^/*μ*L)	(mg/dL)	(U/L)
1	46	2.36	1030
6	81	2.75	780
14	190	5.04	745
21	128	2.99	615
28	149	3.01	448
35	182	2.16	289
42	267	1.86	582
49	261	2.29	284
